# Human-Centered Design of a Multistakeholder Reporting Dashboard for Disseminating HIV Implementation Research in the Ending the HIV Epidemic Initiative: Development and Formative Evaluation Study

**DOI:** 10.2196/92156

**Published:** 2026-07-30

**Authors:** Tyler Scott Johnson, Anna-Sophia Katomski, Reva Datar, Lisa Lucas, Mary Anne Elizabeth Roach, Stefan Baral, Colleen F Hanrahan, Christopher Kemp, Sheree R Schwartz, Laura K Beres

**Affiliations:** 1 Department of International Health, Bloomberg School of Public Health, Johns Hopkins University, 615 N Wolfe St, Baltimore, MD, 21205, United States, 1 410-955-3543; 2 Department of Epidemiology, Johns Hopkins University, Baltimore, MD, United States

**Keywords:** HIV infections, implementation science, health information systems, data visualization, user-centered design, information dissemination, community participation, United States

## Abstract

**Background:**

Interactive dashboards have expanded public access to HIV surveillance and programmatic data, but existing tools do not synthesize implementation research evidence into formats accessible to the diverse stakeholders who could use it. Designing such tools requires deliberate alignment with users’ needs, language, and decision-making contexts.

**Objective:**

We describe the human-centered design (HCD) process used to develop and conduct a formative evaluation of the Ending the HIV Epidemic (EHE) Implementation Science (IS) Reporting Dashboard, a public-facing tool intended to synthesize HIV implementation research conducted in EHE priority jurisdictions, and to identify generalizable lessons on translating IS evidence for diverse academic and nonacademic audiences.

**Methods:**

Guided by the Design Council’s Double Diamond framework, the Johns Hopkins HIV IS Hub used iterative Discover, Define, Develop, and Deliver phases to conceptualize, prototype, and refine the dashboard. Initial consultations with representatives from the National Institutes of Health and a national HIV IS coordination team identified 5 primary user groups: funders, academics, community partners, implementing partners, and domestic HIV IS experts. The Define phase used a purposeful working corpus of 15 EHE IS publications to develop a theory-informed, inductively refined extraction structure. From June 2024 to April 2025, we conducted 14 virtual user-testing sessions across 3 rounds, using purposeful recruitment through established professional networks. Round 1 included 4 academics; rounds 2 and 3 each included 5 participants, with 1 representative from each user group. Sessions used a semistructured dashboard walkthrough, were recorded and transcribed, and were analyzed by 3 team members using rapid qualitative analysis. Notes and transcript observations were organized into structured insight-translation tables and synthesized through team consensus meetings.

**Results:**

The HCD process produced a dashboard concept, a user-group and use-case framework, a data extraction architecture, wireframes, and a prototype organized around study design, populations, outcomes, and community engagement. User testing generated 5 cross-cutting themes: IS terminology created translation barriers; users needed clearer orientation to the dashboard’s purpose and audience; nonacademic users wanted plain-language, action-oriented summaries of research findings; representing community organizations and population affiliations required attention to consent and political sensitivity; and sustained use would require active dissemination beyond launch. These findings informed revisions to navigation, terminology, glossary features, plain-language study profiles, consent-based organization profiles, and dissemination planning.

**Conclusions:**

This study illustrates how HCD can inform both interface design and upstream evidence synthesis decisions when developing dissemination tools for implementation research. We offer practical lessons on translating IS evidence for diverse academic, public health, and community audiences.

## Introduction

Rapidly translating research on effective HIV prevention and treatment interventions into clinical and community practice is essential for improving prevention and treatment outcomes, yet evidence is often underutilized in real-world settings [1,2]. Barriers such as limited access to research outputs, inadequate dissemination strategies, and challenges in engaging diverse stakeholders continue to impede the application of evidence to practice. Formative approaches that actively involve end users in developing implementation tools can improve the relevance, accessibility, and uptake of research findings, with human-centered design (HCD) as one example [3,4]. By focusing on intended users’ goals, decision-making processes, data literacy, and lived experiences, HCD methods use early and ongoing empirical insights from intended users to inform the development of public health interventions and data tools that are more intuitive, accessible, and responsive to stakeholder needs [3-5].

Information dashboards are collections of visual displays that enable users to explore, interpret, and derive insights from complex datasets [6]. Public health agencies have long used dashboards to support syndromic surveillance and inform resource allocation [7], with domestic HIV programs offering prominent examples: America’s HIV Epidemic Analysis Dashboard (AHEAD) supports HIV program monitoring and surveillance activities, and AIDSVu and the Centers for Disease Control and Prevention National Center for HIV, Viral Hepatitis, STD, and TB Prevention AtlasPlus visualize HIV epidemiologic and surveillance data [8-10]. These dashboards have demonstrated the value of interactive data visualization for surveillance, crisis management, interagency coordination, and public communication [5,11]. Existing public health dashboards primarily focus on programmatic, clinical, or epidemiologic surveillance data and less frequently synthesize implementation research evidence to support decision-making among policymakers, practitioners, and community stakeholders.

Despite their potential as dissemination tools, the clarity, relevance, and utility of dashboards are often constrained by poorly defined measures, limited stakeholder involvement, and resource constraints [12]. Users vary widely in their objectives, data interests, visualization literacy, and lived experiences, making generalized design approaches inadequate [6]. Integrating HCD methods into dashboard development helps prevent misalignment with user needs and decision-making contexts by systematically identifying user priorities, informing data presentation, and tailoring functionality to diverse audiences [3,4].

The need for effective, user-informed data tools is especially salient within the Ending the HIV Epidemic (EHE) initiative. The EHE initiative aims to reduce new HIV infections in the United States by 90% by 2030 by scaling up evidence-based prevention and treatment strategies [13]. Achieving this goal requires timely identification of context-appropriate interventions and implementation strategies and the rapid application of emerging data to inform implementation and resource allocation [14]. Dashboards can support these efforts by aggregating, visualizing, and contextualizing data to help policymakers, implementers, and community partners assess trends, identify gaps, and prioritize interventions across EHE priority jurisdictions—48 counties, the District of Columbia, San Juan, Puerto Rico, and 7 states with a high rural HIV burden [15]. Although these jurisdictions account for more than half of new HIV diagnoses, implementation research has been underutilized to guide epidemic response [16].

From 2019 to 2023, the National Institutes of Health (NIH) funded more than 200 EHE Implementation Science (IS) supplement awards to strengthen researcher–community partnerships focused on HIV treatment and transmission [13]. While this investment has generated a substantial evidence base, effectively disseminating and translating findings for diverse stakeholders remains a persistent gap. Dashboards are one potential strategy for organizing and sharing this evidence, though their utility depends on alignment with stakeholder priorities and decision-making contexts.

This study presents a formative HCD investigation that informed the design and refinement of the EHE IS Reporting Dashboard, an online tool that disseminates HIV-related implementation research to stakeholders advancing the EHE initiative. Specifically, we aim to describe the iterative HCD process by which the dashboard’s content, structure, and functionality were defined and refined, and to identify generalizable lessons on translating IS evidence for diverse nonacademic audiences via interactive dashboards.

## Methods

### Study Design

We developed a human-centered, publicly accessible, and regularly updated dashboard that summarizes and synthesizes domestic HIV implementation research conducted within EHE priority jurisdictions and aligned with EHE goals and objectives. Translating evidence into action beyond the individual study level depends on broad access to study results, as well as on transferability and generalizability. Achieving the goals of the EHE initiative requires understanding outcomes at a national scale, which, in turn, relies on the ability to rapidly assess data across studies and geographies. Therefore, to conceptualize and operationalize the product, we employed an iterative, reflexive development process grounded in the Design Council’s Double Diamond framework.

### Setting and Time Period

The core dashboard development team (hereafter, “the team”) comprised faculty members of the Johns Hopkins University IS Hub (formerly the Mid-Atlantic Centers for AIDS Research [CFAR] Consortium IS Hub), full-time research associate staff, PhD/DrPH students, postdoctoral fellows, and a professional web developer. The Johns Hopkins University IS Hub is one of several NIH-funded consultation entities that provide technical assistance and support capacity-building to strengthen domestic HIV implementation research funded by the NIH. NIH funding supported the design and development of this dashboard as part of its mandate to advance IS tools and resources for the EHE initiative.

Discover-phase consultations were conducted between November 2022 and January 2023. Define-phase publication identification and extraction work were conducted between March and July 2023. Develop-phase wireframing and prototyping occurred between September 2023 and June 2024. Deliver-phase user-testing sessions were conducted between June 2024 and April 2025.

### Conceptual Framework

We used the Design Council’s Double Diamond framework to guide the dashboard development process across 4 iterative phases: Discover, Define, Develop, and Deliver [17]. The Discover phase sought to understand the landscape of potential end users and identify the types of data and visualizations most valuable for decision-making, with the objective of defining the dashboard’s overall scope and purpose. The Define phase translated user priorities into actionable design requirements and data specifications, clarifying what the dashboard needed to do and how it could best communicate findings to distinct user groups. In the Develop phase, we translated conceptual requirements from the Discover and Define phases into tangible design elements, which were then synthesized into a prototype of the dashboard’s structure, navigation, and content. During the Deliver phase, we evaluated the dashboard’s usability and relevance through structured user testing and iterative feedback.

### Discover Phase

Faculty members conducted initial consultations with representatives from the NIH and an NIH-funded national HIV IS coordination team [18]. These stakeholders were uniquely positioned to support coordination across EHE IS studies and to encourage the use of cross-study resources to advance the field of IS while supporting the rapid achievement of EHE goals. Meetings focused on presenting the initial dashboard concept for feedback and discussing proposed implementation research characteristics and variables for inclusion. The team synthesized insights from the initial consultations to produce a core dashboard concept, a set of defined user groups, and preliminary use cases.

### Define Phase

We identified a working corpus of IS-focused EHE publications (n=15) to guide activities throughout the Define phase. A colleague from the NIH-funded HIV IS Coordination Initiative provided the team with an initial list of 22 EHE publications that EHE investigators had self-reported as grant-related outputs. To support the linkage of publications to specific EHE projects, the Coordination Initiative also provided project descriptions that included the project title, CFAR affiliation, principal investigator, project director, EHE jurisdiction(s), and community or implementing partner. Of the 22 self-reported publications, 15 met the eligibility criteria for inclusion: original research articles that could be linked to a specific EHE project. This purposeful working corpus was intended to support tool development rather than serve as a systematic review of the published EHE IS literature.

We identified variables for data extraction using a hybrid deductive-inductive approach. Deductively, we drew on Hickey et al [19] specification of HIV implementation interventions, the Consolidated Framework for Implementation Research for determinants [20], and Proctor et al [21] taxonomy of implementation outcomes. Inductively, we reviewed the 15 publications to identify emergent variables and data elements that captured how implementation was described in practice. We iteratively refined the variables through team consensus meetings during extraction, drawing on patterns observed across publications and the team’s collective expertise in HIV IS. We then organized these variables into a structured online database (Airtable [22]) that served as the data extraction tool.

Five team members (TSJ, ASK, RD, LL, and LKB) conducted extractions from the initial set of publications. Each record’s data were first extracted by 1 team member and then independently reviewed by a second for accuracy and completeness. Any discrepancies identified during the second review were resolved through group consensus. The online extraction tool was iteratively refined throughout the process and ultimately grouped data into the categories of study design, study population, outcomes, and community engagement.

Following the initial extractions, the team held internal consultations to map available data to the use cases identified in the Discover phase. We aligned each use case with specific user queries and priority variables to predict how distinct user groups might interact with the dashboard and answer questions most relevant to their roles and positionality within the broader EHE initiative. The consultations also clarified how the data architecture might inform visualization design and filtering options to answer user-specific questions.

### Develop Phase

We created low-fidelity wireframes to demonstrate how the extracted data might be visualized to both leverage their opportunities and manage their limitations in meeting the dashboard’s objectives and use cases. We conducted a “storyboarding” exercise to visualize a hypothetical user’s journey through the website, considering how the data might be grouped to be accessible and informative for all users while still distinguishing among user types. We used PowerPoint and physical drawings to imagine which data would be available on each page and how they might be visualized. We shared the wireframes with an external collaborator, who produced a low-fidelity Python prototype. Both the wireframes and the prototype were used to facilitate the initial round of user testing.

### Deliver Phase

We conducted 14 user-testing sessions with EHE stakeholders across 3 rounds. Sessions were facilitated by members of the research team (LKB and SRS: professors, PhD; RD: postdoctoral researcher, PhD; TSJ: DrPH candidate, MPH; ASK: research manager, MPH), all trained in qualitative methods. Participants were selected primarily by user group affiliation; candidate recruitment discussions also considered proximity to EHE implementation, geographic experience across EHE priority jurisdictions, and familiarity with IS. Participants were known professional contacts of the research team before recruitment; invitation materials described the project as developing a dashboard to synthesize NIH-funded EHE IS research and framed the sessions as consultations rather than formal research. Formal demographic data were not collected, given the nonhuman subjects institutional review board determination; all participants were selected based on at least a decade of relevant experience within their respective user-group domain. No invited participants declined or withdrew.

One team member facilitated each 60-minute session; a second was present to take notes and provide supplementary facilitation as needed. No additional nonparticipants were present. Sessions were conducted virtually via Zoom, recorded, and transcribed. Sessions began with a guided walkthrough of the prototype, accompanied by live feedback from the participant. Participants then responded to embedded semistructured questions on both function (practical interaction/logistics) and form (content), including how they might use the dashboard in daily work and how accessibility and usability might be improved. Each session was intentionally structured to mirror the stages of the Double Diamond framework, guiding participants through questions that sequentially reflected the Discover, Define, Develop, and Deliver phases, and allowing each session to function as its own nested HCD cycle (Multimedia Appendix 1).

### Analysis

We used a rapid qualitative analysis approach to synthesize session data [23,24]. During each session, one team member captured real-time notes to support in-session probing; session transcripts were later reviewed to inform postsession synthesis. Notes and transcript observations were organized into a structured spreadsheet with columns for dashboard sections and elements, user observations, and suggested adaptations. Three team members (ASK, LKB, and TSJ) led the analysis; findings from each session were synthesized across all sessions within a given round during team consensus meetings, where interpretive discrepancies were discussed and resolved. This formative study was not designed to achieve thematic saturation; the sample size was determined a priori to ensure representation of all planned user groups in each testing round [23]. Themes were derived inductively from the structured templates through team consensus; no prespecified coding framework or formal coding tree was used, consistent with the rapid qualitative approach [23]. Synthesized findings were converted into actionable recommendations for the external web developer, covering both microlevel interface adjustments (such as terminology, navigation, and layout) and macrolevel conceptual shifts, including user tailoring, data transparency, and dissemination features.

### Ethical Considerations

This research project was reviewed by the Johns Hopkins Bloomberg School of Public Health Institutional Review Board and received a nonhuman subjects research determination. Verbal consent for recording and the use of deidentified feedback was obtained at the start of each session. Community partner participants received a US $50 gift card in recognition of their time.

## Results

### Discover Phase

Consultations and team synthesis produced 3 primary outputs: a core dashboard concept, a defined set of user groups, and an initial set of use cases.

The envisioned product was a publicly accessible, interactive dashboard for reviewing and summarizing published EHE implementation research studies. The dashboard was intended to tailor content to distinct user priorities, offer visualizations of implementation research characteristics, and represent qualitative work, which was a significant part of the published EHE research. Importantly, this new dashboard was conceived as research outcomes–focused, in contrast to existing dashboards that primarily provide programmatic updates.

We identified 5 primary user groups: (1) the project’s funders, the NIH; (2) academics; (3) community partners, such as nongovernmental organizations and community-based organizations; (4) implementing partners, including local and state health departments; and (5) experts in HIV-related IS in the United States. For each stakeholder group, we used data from consultative meetings to answer three questions: (1) what might interest a representative of that group about the dashboard; (2) what questions might that individual ask about published EHE research results; and (3) how can the dashboard meet that individual’s needs? (Table 1).

**Table 1.** Identified user groups for the Ending the HIV Epidemic Implementation Science Reporting Dashboard and their associated interests, priorities, and needs.

Stakeholder group	What might interest them about the dashboard?	What questions might they ask about EHE^a^ implementation research?	How might the dashboard help meet their needs?
NIH^b^	Reinforcing investment to Congress	Which geographic areas have funded projects? How many participants have engaged in EHE-funded projects? Is research focused on implementation, clinical, or service outcomes?	Study frequency heat map Detailed numbers and demographic information of study participants
NIH	Mapping investments to strategic planning goals	What work has been done under each strategic focus area?	Organize studies/outcomes by strategy (ie, diagnose, treat, prevent, and respond)
NIH	Analyzing intersectional demographic data of participants	What studies include priority populations, such as Black MSM^c^?	Priority populations tab; tick boxes for different demographic groups
Academics	Identifying new research questions	Do implementation outcome results illuminate hypothesis-generating patterns across studies?	Grouping of studies by outcome
Academics	Creating a sampling frame for research	What/how many participants are in each study? Who should be sampled for future implementation studies?	Participant data Study design tab
Academics	Answering metascience questions	How are IS^d^ frameworks currently applied in practice?	Filter dashboard by IS framework
Academics	Identifying gaps in research	Where do gaps in implementation research exist?	Gaps in the heat map (ie, geographic areas with no/fewer publications) Gaps in studies appearing when various filters are applied throughout the site
Community partners (eg, NGOs^e^, CBOs^f^)	Identifying new/improved strategies to implement interventions	What have we learned to help us better implement PrEP^g^? Who is working with Black women? What are barriers and facilitators to rapid ART^h^? Where can I learn more?	Filter by population/intervention Link to papers cited in the dashboard
Community partners (eg, NGOs, CBOs)	Connecting with other NGOs/CBOs	What other organizations are working in my geography?	Zoom in on the relevant EHE priority jurisdiction and view all information
Domestic HIV-IS experts	Gaining insights into community engagement practices and community partnerships	What do community partnerships look like in different regions of the country? How many and what types of community partners have been engaged?	Community engagement tab
Implementing partners (eg, BCHD^i^)	Identifying priority areas for future funding allocation	Which approaches seem to be effective? What seem to be ongoing challenge areas?	Barriers/facilitators tab Implementation outcomes tab
Implementing partners (eg, BCHD)	Monitoring and evaluating existing projects	What have other programs done that work well? How can we improve our existing programming?	Barriers/facilitators tab Implementation outcomes tab

a EHE: Ending the HIV Epidemic.

b NIH: National Institutes of Health.

c MSM: men who have sex with men.

d IS: implementation science.

e NGO: nongovernmental organization.

f CBO: community-based organization.

g PrEP: pre-exposure prophylaxis.

h ART: antiretroviral therapy.

i BCHD: Baltimore City Health Department.

We then used insights from Table 1 to identify 5 primary use cases that would guide the dashboard’s design: (1) collate published EHE IS data into a single repository; (2) support rapid review of the current “state of the (implementation) science”; (3) provide accessible data to answer implementation questions; (4) facilitate the generation of research questions and the development of sampling frames for EHE-related systematic reviews and other evidence syntheses; and (5) facilitate partnership and network development by identifying academic and implementation researchers.

### Define Phase

The Define phase translated the 5 use cases identified in the Discover phase into concrete data requirements and a structured information architecture for the dashboard. The team mapped each use case to the types of user queries it would need to support and the specific variables that would need to be captured to answer those queries (Table 2). This process clarified both what data would need to be extracted from the published literature and how the resulting database would need to be structured to support filtering and visualization across user groups. Extraction activities ultimately grouped variables into 4 primary domains—study design, study population, outcomes, and community engagement—forming the backbone of the dashboard’s data architecture.

**Table 2.** Primary use cases, example user queries, and priority variables informing dashboard design.

Use case	Example user query	Priority variables
Collate published EHE^a^ implementation science data into a single repository	What EHE research has been published?	Article title Authors Journal title Year of publication
Support rapid review of the current “state of the (implementation) science”	Have EHE IS^b^ studies improved our understanding of the utility of X framework?	Type of result (implementation outcomes, determinants) Specific barriers/facilitators IS frameworks
Provide accessible data to answer implementation questions	What have we learned about barriers and facilitators to different interventions?	Specific barriers/facilitators Design type Evidence-based intervention Type of result (implementation outcomes, determinants)
Facilitate research question generation and sampling frame development for EHE-related systematic reviews and other evidence syntheses	What do the data tell us in aggregate?	Type of result (implementation outcomes, determinants) Community partnerships Priority populations
Facilitate partnership and network development through identification of academic and implementer researchers	Who is working on EHE research and implementation?	Community partners Implementing partners EHE priority jurisdictions Type of community partner

a EHE: Ending the HIV Epidemic.

b IS: implementation science.

### Develop Phase

**Figure 1.**
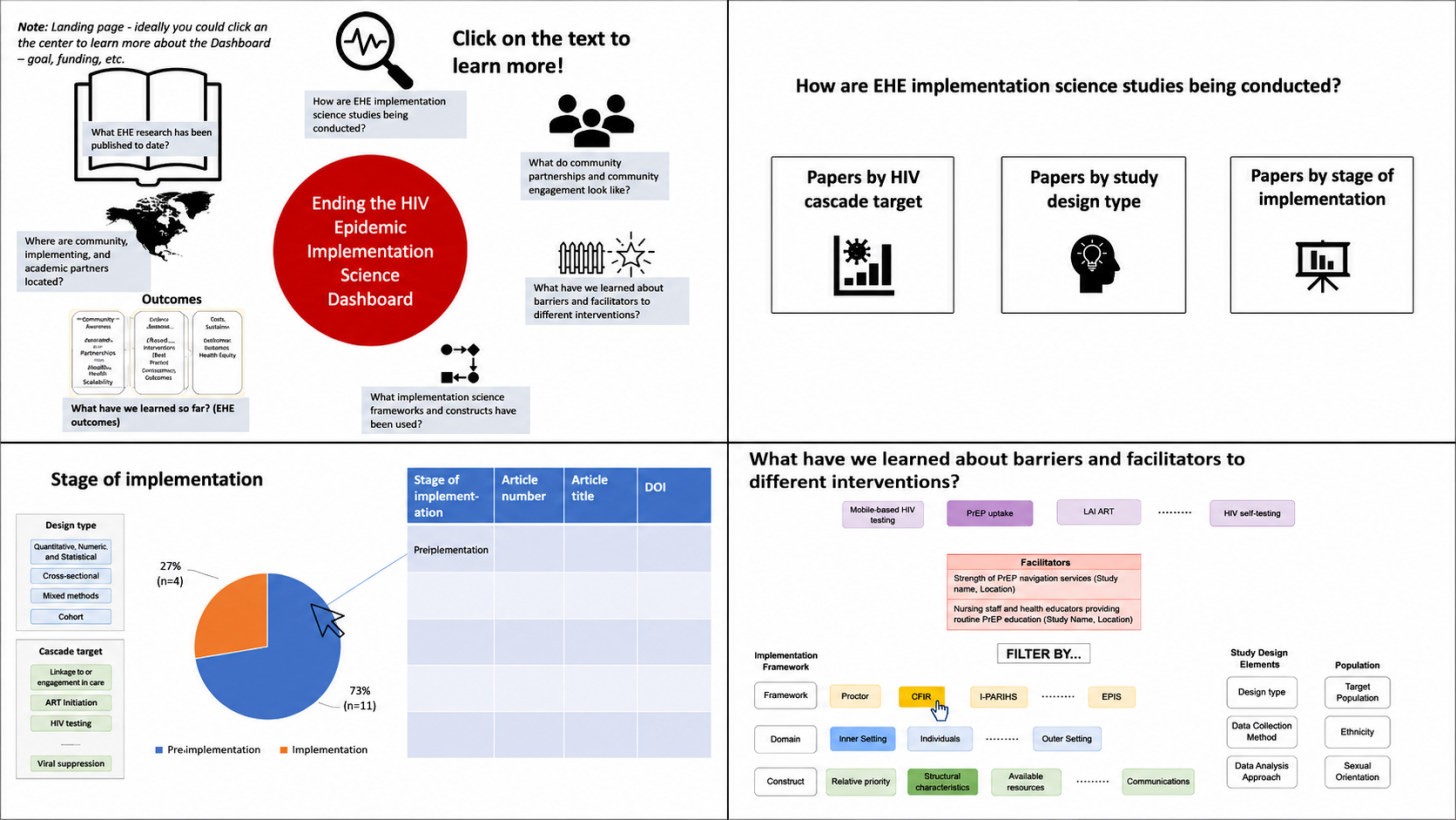
Low-fidelity wireframes illustrating the preliminary structure and key pages of the Ending the HIV Epidemic Implementation Science Reporting Dashboard. Each panel represents an initial conception of a page that would eventually be integrated into the dashboard prototype by a professional web developer. The wireframes were informed by data and insights synthesized throughout the Discover and Define phases of the dashboard development process. Top left: the landing page, in which user queries were oriented around a central navigation button; top right: options that would guide users to specific visualizations based on predetermined data filters; bottom left: brainstorming the structure of a specific visualization, with associated filters and a publication table that would populate based on user selections; bottom right: preliminary conception of the “Barriers and Facilitators to Implementation Success” page. ART: antiretroviral therapy; CFIR: Consolidated Framework for Implementation Research; EHE: Ending the HIV Epidemic; EPIS: Exploration, Preparation, Implementation, Sustainment; i-PARIHS: integrated Promoting Action on Research Implementation in Health Services; LAI-ART: long-acting injectable antiretroviral therapy; PrEP: pre-exposure prophylaxis.

The Develop phase produced 3 outputs: low-fidelity wireframes and storyboarded user journeys; a Python-based prototype that integrated priority variables and views; and an initial information architecture specifying page types, navigation, and filters aligned with the Discover and Define insights. The wireframes included mock-ups of a landing page, a table summarizing research to date, maps of EHE project and community partner locations, and pages for barriers and facilitators, IS frameworks, implementation outcomes, client outcomes, service outcomes, HIV cascade targets, and study designs (Figure 1).

### Deliver Phase

#### User Testing

The first round of testing included 4 academics. The second and third rounds each included 5 participants, 1 from each of the 5 user groups identified during the Discover phase (funder, academic, community partner, implementing partner, and domestic HIV IS expert).

Each round of user testing revealed specific design issues and corresponding adaptations. Table 3 presents an illustrative summary of the dashboard elements discussed, the user insights generated, and the adaptations or next steps prompted by those insights.

Beyond the element-level adaptations, we identified 5 cross-cutting themes that recurred across user groups and rounds. These themes are illustrated with paraphrased observations from the session notes and transcripts.

**Table 3.** Examples of insight translation from user testing to dashboard adaptations for the Ending the HIV Epidemic Implementation Science Reporting Dashboard.

Dashboard element	Insight	Adaptation/next step
Landing page—general	Users found the homepage visually appealing but unclear in purpose; several asked, “What is this, and who is it for?”	Added a brief description of the dashboard’s mission and intended audiences; included a short explanation of the EHE^a^ initiative.
Landing page—navigation	Users were uncertain where to begin and requested clearer entry points.	Reordered and renamed tabs for intuitive flow (eg, “About,” “Publications,” “Data,” and “Community”), added a search bar, and developed a short interactive tutorial for first-time users.
Landing page—design	The layout was viewed as busy, and users wanted to tailor content by role (eg, community partner vs academic).	Planned role-based landing page options (“Show me everything,” “I’m a researcher,” and “I’m a community partner”) and streamlined tiles to reduce redundancy.
Barriers and facilitators page	Users interpreted “barriers” as client-level rather than implementation-level obstacles.	Renamed the tab to “Barriers and Facilitators to Implementation Success” and added a short page description to clarify scope.
Filters and terminology	Terms such as “Cascade Target” and “Study Population” caused confusion among nonacademic users.	Simplified terminology (eg, “Continuum Target,” and “Study Participant Classification”) and added glossary links and hover definitions.
Publications table and map	Users wanted to interact with the map directly and felt the publication table lacked context.	Enabled clickable filtering by jurisdiction, clarified column labels, and added a one-line explanation of what the map represents.
Community partnerships map	Users expressed concern about listing organizations without consent and about sensitive population affiliations.	Added a consent-based intake process and an option for organizations to select which information appears publicly.
Implementation frameworks visualization	Displaying the frequency of frameworks was seen as overwhelming and unclear in purpose.	Simplified visuals, separated framework graphs into their own page, and added brief explanatory text describing their intended use.
Glossary and tutorials	Users requested easier access to definitions and orientation materials.	Added embedded glossary links that open in small pop-up windows and expanded tutorial prompts on key pages.
Dissemination and engagement features	Several users suggested a space for sharing new findings and increasing community visibility.	Proposed creation of a new “Conversation” tab featuring emerging findings, conference materials, and study updates to foster ongoing dissemination and collaboration.

a EHE: Ending the HIV Epidemic.

#### Theme 1: IS Terminology as a Translation Barrier

Across multiple sessions, terms widely used in IS were interpreted by community and implementing partners, and even some academic participants, in ways that diverged from the team’s intended meaning. Most consistently, “barriers and facilitators” was understood to refer to individual-level obstacles or supports for clients rather than to system- or implementation-level determinants of intervention delivery. Similar interpretive drift was observed for “cascade target,” “study population,” and the EHE “pillars” framing, with one participant noting that unless a user is very research-oriented, they would likely think in terms of the HIV cascade rather than pillars. Acronyms such as Consolidated Framework for Implementation Research, Exploration, Preparation, Implementation, Sustainment, and long-acting injectable antiretroviral therapy were also flagged as inaccessible to non-IS audiences. The team responded by renaming the affected dashboard tab to “Barriers and Facilitators to Implementation Success,” replacing “Cascade Target” with “Continuum Target,” removing the “Study Population” filter, and adding a glossary with hover definitions throughout the dashboard.

#### Theme 2: Orientation and Purpose Were Unclear at First Contact

Nearly every participant asked, in some form, “What is this, and who is it for?” upon arriving at the landing page. Participants reported feeling neither oriented to the dashboard’s mission, its intended audience, nor how to begin navigating it. Participants from all 5 user groups asked different initial questions upon first viewing the dashboard. Researchers asked about study design and outcomes; community partners asked about populations and locations; and implementing partners asked about practical takeaways. In response, the team added a clear “About” section as the first navigation tab, included EHE context (start date, 2030 goal, and definition of priority jurisdictions) on the landing page, and developed an interactive first-use tutorial.

#### Theme 3: Demand for Plain-Language, Action-Oriented Translations of Academic Content

Community and implementing partner participants consistently reported that academic abstracts lacked sufficient plain-language information to support practical decision-making. Several requested short summaries that prioritized what was tested, where, with whom, and what was learned. Across user groups, participants suggested additional layers of translation: plain-language summaries alongside abstracts, “key takeaways” or “research and practice implications” for each study, and multiple stakeholder-specific topline messages per study. Participants also suggested allowing study authors to link to their own user-friendly materials (eg, websites, protocols, and social media templates) to connect users with those who can provide implementation-level detail beyond what publications provide. The team responded by developing plain-language study profile summaries, planning “key takeaway” fields for each study, and proposing an author-contributed materials feature.

#### Theme 4: Ethical and Political Risks of Representing Community Organizations and Populations

Multiple participants raised concerns about listing community organizations on the dashboard without their explicit consent, especially when an organization’s work could imply affiliation with a politically targeted population. The team responded by designing a consent-based intake process that allows organizations to control what information appears on their public profiles, removing the “Study Population” filter from the publications table to avoid exposing politically sensitive population categories, and adding disclaimers about how organizations are identified and included.

#### Theme 5: Building an Audience Requires Active, Ongoing Dissemination Beyond Launch

A finding that emerged consistently across rounds was that a dashboard, on its own, does not drive use. Participants in every round emphasized that sustained engagement would require active dissemination strategies (eg, promotion through CFAR networks, professional listservs, NIH/Centers for Disease Control and Prevention/Health Resources and Services Administration channels, and conference presentations) and that one-time exposure (eg, via QR codes at conferences) rarely produced return visits. Several participants emphasized the need for a regular cadence of fresh content to give users a reason to return, and one participant proposed monthly thematic spotlights, “what’s new” features, and showcases of how users had applied dashboard insights. Participants also noted the value of differentiating the dashboard from existing tools (AHEAD, AIDSVu, and the ISCI [Implementation Science Coordination Initiative] dashboard) to clarify its specific value proposition. The team responded by proposing a new “Conversation” tab to host emerging findings, conference materials, study spotlights, and provisional results between publication cycles, and by planning a launch dissemination strategy that includes listserv outreach, social media engagement, and a launch webinar.

The dashboard is available online [25]. The ongoing population of the dashboard with newly published EHE IS studies continues.

## Discussion

This study describes the development and iterative testing of an interactive dashboard designed to synthesize and disseminate HIV implementation research to advance the EHE initiative. Across the four Double Diamond phases, design decisions were progressively shaped by consultations and structured user testing rather than being predefined by the design team. The Discover and Define phases produced a framework of 5 user groups and 5 use cases, along with a theory-informed extraction architecture. The Develop and Deliver phases used iterative wireframing, prototyping, and 14 user-testing sessions to translate feedback into interface refinements and improvements in the synthesis and presentation of evidence. Each user-testing session was structured as a nested HCD cycle, allowing the same iterative logic to operate within and across rounds. We synthesize these methodological lessons learned for other teams developing dissemination tools for implementation research.

This study presents a formative research case that illustrates how HCD can be applied not only to the design of a digital tool’s interface but also to the upstream processes of evidence synthesis, terminology selection, and audience tailoring, which determine whether the tool reaches its intended audience. Most published HCD case studies in health focus on patient-facing tools or single-intervention applications; we extend this literature by applying HCD to a cross-study evidence-synthesis tool intended to serve heterogeneous stakeholder groups within a national public health initiative. Our findings on terminology, audience tailoring, community-organization representation, and the limits of academic dissemination conventions provide concrete material for other teams developing similar dissemination tools.

Several lessons from our process are relevant to teams developing similar implementation-focused dashboards. Terminology review should occur early in the design process. We found that field-standard terms are interpreted differently by the community and implementing partners and that translation problems embedded in navigation or filters are harder to correct later. Plain-language summaries, stakeholder-specific framing, and links to author-contributed materials can enhance usability for nonacademic audiences. Further, a single neutral landing page rarely serves heterogeneous user groups well; role-based entry points, prominent orienting content, and first-use tutorials reduce the burden on users who arrive without prior context. Taken together, these considerations reflect a broader principle: translating implementation research for academic and nonacademic audiences is a commitment that requires substantial resources, time, and technical infrastructure from the outset. In our case, this required a third-party web development contract, which was essential to producing a tool that could be maintained and updated over time.

Beyond content, we found that listing community organizations on a public dashboard is not equivalent to displaying study results and that consent options giving organizations control over their public representation are important both ethically and practically. Finally, building a sustained audience for a dissemination tool requires active dissemination, planned as part of its development. Participants viewed the dashboard’s existence as the start of dissemination work, not its completion.

Our experience extends the existing literature on public health dashboards in several ways. Schulze et al [5] systematic review of public health data dashboards identified visualization clarity, stakeholder involvement, and resource availability as recurring constraints; our process illustrates how a HCD approach can address each of these within a single tool. Our terminology-related findings are consistent with the broader knowledge translation literature documenting splits between academic and community framings of health concepts [11,26]. We note that this gap persists even for vocabulary developed specifically to bridge research and practice (eg, “barriers” and “facilitators”), suggesting that translational language in IS may itself require translation for the practitioners it aims to reach. The demand for plain-language summaries and stakeholder-tailored content aligns with prior dissemination research showing that academic formats underperform without adaptation for nonacademic audiences [11,27] and extends that research by specifying that research-synthesis tools may require layered translations, such as plain-language summaries and stakeholder-specific framings. The ethical and political concerns that participants raised about representing community organizations align with the community-engaged research literature on consent and partner control over public representation [28], a concern that is particularly urgent in the current US political context for organizations serving targeted populations. Our finding that active, ongoing dissemination is required for sustained use aligns with longstanding distinctions between active and passive dissemination in IS [27]; our process adds that user testing itself identified this as a design requirement rather than a postlaunch afterthought. Finally, to our knowledge, the use of nested HCD cycles within user-testing sessions has not been previously described in the IS tool development literature [4,29].

Several limitations of our approach warrant consideration. First, the structured insight-translation framework we used to capture cross-cutting themes emerged later in the process rather than at the outset; earlier formalization might have reduced the risk of overlooking patterns during the early rounds of testing. Second, our reliance on an external web development contractor introduced challenges related to continuity and content familiarity; external partners did not always have deep familiarity with the underlying IS content, occasionally requiring additional time to align on priorities and feasibility. Third, user testing focused on a purposive sample of stakeholders within the research team’s established professional networks. Participants were largely known professional contacts, which may have shaped which voices were accessible and how feedback was offered. Social desirability and politeness norms common in collegial professional networks may have moderated criticism, particularly from community and implementing partner participants. We sought to mitigate these dynamics through structured prompts that explicitly invited critical feedback, anonymization of session notes during synthesis, and modest US $50 gift card compensation for community partner participants; nevertheless, the team’s institutional positioning through its affiliation with an academic institution and a NIH-funded IS Hub likely shaped both who was reached and how the dashboard’s overall framing was constructed. Fourth, this study describes a single team’s experience designing a single dashboard; we do not compare HCD with alternative approaches and so cannot make comparative claims about HCD’s relative effectiveness. The study should be read as a worked illustration of an HCD-guided process, not as an evaluation of HCD as a method.

Applying HCD throughout the design and development of the EHE IS Reporting Dashboard supported continuous alignment with stakeholder needs and yielded transferable lessons on translating IS evidence for nonacademic audiences. Treating HCD as ongoing infrastructure for formative evaluation, rather than a one-time design step, allowed both interface and content choices to be reshaped in response to user feedback. These lessons may inform the development of future tools that seek to bridge the gap between implementation research and real-world decision-making.

## Supplementary material

10.2196/92156 Multimedia Appendix 1 Question guide.

## Abbreviations

AHEAD: America’s HIV Epidemic Analysis Dashboard
CFAR: Centers for AIDS Research
EHE: Ending the HIV Epidemic
HCD: human-centered design
IS: implementation science
ISCI: Implementation Science Coordination Initiative
NIH: National Institutes of Health
