# Supplementary material for: Human-Centered Design of a Multistakeholder Reporting Dashboard for Disseminating HIV Implementation Research in the Ending the HIV Epidemic Initiative: Development and Formative Evaluation Study
Source: JMIR Form Res. 2026 Jul 30;10:e92156. doi: 10.2196/92156 (PMC13422908; doi:10.2196/92156)
Supplement: Multimedia Appendix 1 [file formative-v10-e92156-s001.pdf]

Appendix. Question Guide

Version Date: 10 April 2025

I. Intro

a. Hello, thank you for joining us today. My name is XX and I will be facilitating the session today. XX will be taking notes. [CLICK]

b. The objective of this work is to create a publicly accessible, interactive dashboard to review and summarize published Ending the HIV Epidemic (EHE) implementation science studies that effectively meets the needs of priority user groups. These priority groups include: 1) EHE community partners, e.g., NGOs and CBOs; 2) implementing partners, e.g., health departments; 3) academics; and 4) the National Institutes of Health (NIH).

c. We have created a version of the dashboard that has some of the basic structure of what we envisioned based on initial discussions with people who may want to use this dashboard. Some of this has been coded into live pages on a website and some of it we have as static mockups. We have spoken with others prior to this to create envisioned use cases and will be speaking with other user groups following today's call. [CLICK]

d. The process for the interview today will involve showing you elements of the dashboard and asking for your thoughts. The intention of the interview is to improve the concept and the form of the final website, so we would love to hear any thoughts you might have on what you like, dislike, and would change.

e. To clarify, this is a consultation, not formal research. We are hoping to record the session to review your feedback as we work with a web developer to finalize the dashboard. Before we proceed, we want to confirm that you are comfortable with us recording this session.

II. Walk Through

Present each component of the dashboard and get feedback component by component using probes.

a. [Landing page - Website or PowerPoint Slide]

This is the landing page for the dashboard. It is planned to be the first thing that users see and provide a user-friendly space to navigate to the pages that the user is most interested in. Let me show you how we envision this working. The center button would allow users to learn more about the purpose of the dashboard, while the other buttons would represent different ways that users could investigate the data. [Read each of the questions]

1. What would your initial thoughts be if you arrived here after clicking on the Dashboard link?
2. Do you find this layout intuitive?
3. Do you have any questions about this? Anything you like or dislike?

b. [EHE Publications by State / EHE Map - Website or Live Demo]

This page shows a geographical display of EHE priority jurisdictions and allows users to visualize where studies have been conducted so far. [WALK THROUGH PAGE AD HOC]

1. More general probe: What do you think? / What do you like or dislike? / How would you improve this?
2. What do you think of the layout of this page?
3. What do you like/dislike about the study information displayed? Is anything missing? Are title and URL sufficient?

c. [Barriers and facilitators - Website or User Demo]

This page shows barriers and facilitators to implementation of various evidence-based interventions. The plan is to display one table for barriers and another for facilitators in the center of the page, and then

have options on the side that allow users to filter barriers and facilitators by other variables. [CLICK MOBILE-BASED TESTING, PROCTOR, ACCEPTABILITY] As you can see, some of these data are not yet complete. We are asking for your feedback on basic usability to inform the completion of the dashboard. I have put a link to this page in the chat for you to take a moment to explore. If you do not mind sharing your thoughts out loud as you explore, that would be great. Then I have some additional questions.

    - Link: https://jhuehedashboard.dr3.tech/barriers-facilitators-2/

- More general probe: What do you think? / What do you like or dislike? / How would you improve this?
- Which additional variables would you want to filter by?
- What, specifically, do you like/dislike about the structure of the table(s)? Of the filters?
- Barriers and facilitators to what? Does this resonate with you as linking it to an intervention?

d. [Community Partnerships - Mockups or PowerPoint Slides]

This page will allow users to visualize where EHE partners are working. We have three sub-pages for this section: one is a map showing where community and implementing partners are located across the country, another assesses community partnerships, and another assesses community engagement.

1. Community Partnerships Map

a. Would you imagine using a map like this in your work? How might you use it?

b. More general probe: What do you think? / What do you like or dislike? / How would you improve this?

c. What specifically do you like/dislike about the community partnership information that is displayed?

2. Community Partnerships

a. What do you take away from this page?

b. What do you think? / What do you like or dislike? / How would you improve this?

c. What do you think about this community partnership rating scale?

- Key et al. 2018, Continuum of Community Engagement in Research: https://muse.jhu.edu/article/743598

3. Community Engagement

a. What do you take away from this page?

b. What do you think? / What do you like or dislike? / How would you improve this?

c. What do you think about this community engagement rating scale?

- Khodyakov, Dmitry, et al. "On measuring community participation in research." Health Education & Behavior 40.3 (2013): 346-354. https://www.ncbi.nlm.nih.gov/pmc/articles/PMC3665736/

e. [Implementation Outcomes - Live Demo]

This page shows implementation outcomes cited in EHE publications. It is planned to allow users to visualize the frequency of various outcomes across all studies and filter by outcome to see more specific data about that outcome. [CLICK ON BAR] We plan to have functionality that would show the list of studies measuring the outcome and provide links to their DOIs, as well as additional information about outcomes measurement once publication of these data is more robust.

1. More general probe: What do you think? / What do you like or dislike? / How would you improve this?
2. [CLICK ON BAR] What, specifically, do you like/dislike about the filter feature? Which information would you want to see when filtering by a specific implementation outcome?

f. [Implementation Frameworks - Website, Mockup, or Live Demo]

This page shows implementation frameworks cited in EHE publications. It is planned to let users visualize the frequency of various frameworks across all studies and filter it based on EHE cascade targets.

1. More general probe: What do you think? / What do you like or dislike? / How would you improve this?
2. What, specifically, do you like/dislike about the filter feature? Which information would you want to see when filtering by a specific EHE target?

g. [How are EHE implementation science studies being conducted - PowerPoint Slide / Live Demo]

The following pages show key study design information related to EHE publications. It is planned to let users filter studies by the HIV cascade target of interest, the design type employed, and the stage of implementation.

[Cascade Target - PowerPoint Slide]

[Design Type - Live Demo] [CLICK ON IMPLEMENTATION PERIOD FILTER]

[Implementation Stages - Live Demo] [CLICK ON STUDY TITLES TO SHOW CHANGE IN DOI LINKS, FILTER GRAPH BY IMPLEMENTATION STAGE]

1. More general probe: What do you think? / What do you like or dislike? / How would you improve this?
2. What, specifically, do you like/dislike about the filter feature?
3. Do you think it is necessary to separate these components into separate pages, or could they be combined into one page?

III. Semi-structured questions

a. Thinking about your role as a XX, how might you use this dashboard? Can you think of any projects or activities that you currently engage in that could utilize this dashboard? How might you or they use it?

b. Any other ideas for identifying publications?

c. Which of these dashboard use cases, if any, would be of interest to you? [present as a PowerPoint slide]

- i. Highlighting published EHE IS data, i.e., manuscripts and conference presentations.
- ii. Supporting rapid review of the state of the science / EHE research outcomes to date.
- iii. Providing accessible data to answer meta-science questions.
- iv. Sampling frame development for EHE-related systematic reviews to support research question generation and other syntheses/gap identifications.
- v. Supporting identification of possible partnerships / network development through identification of academic and implementer researchers.

d. Our anticipated user groups are: [present as a PowerPoint slide]

Staff members at the National Institutes of Health, academics, community partners (e.g., NGOs, CBOs), leadership of the Implementation Science Coordination, Collaboration, and Consultation Initiative (an NIH-funded body), and implementing partners (e.g., city/county health departments). Can you think of anyone else who might have interest in using the dashboard?

e. For people within your network [user group], what might be ways to:

- a. Let them know this dashboard exists?
- b. Promote access of the dashboard?
- c. Improve use?

f. Any other thoughts or things you would like to share?
